# Core Values that Influence the Patient—Healthcare Professional Power Dynamic: Steering Interaction towards Partnership

**DOI:** 10.3390/ijerph17228458

**Published:** 2020-11-15

**Authors:** Angela Odero, Manon Pongy, Louis Chauvel, Bernard Voz, Elisabeth Spitz, Benoit Pétré, Michèle Baumann

**Affiliations:** 1 Department of Social Sciences, Institute for Research on Sociology and Economic Inequalities (IRSEI), University of Luxembourg, 4365 Esch-sur-Alzette, Luxembourg; angela.odero@uni.lu (A.O.); manon.pongy@uni.lu (M.P.); louis.chauvel@uni.lu (L.C.); 2 Department of Health Psychology, Metz Saulcy Campus, University of Lorraine, 54395 Grand Est, France; elisabeth.spitz@univ-lorraine.fr; 3 Department of Public Health, University of Liège, 4000 Liège, Belgium; bernard.Voz@uliege.be (B.V.); benoit.petre@uliege.be (B.P.)

**Keywords:** patient-as-partner, power, patient empowerment, chronic diseases, patient participation, qualitative analysis, NVivo, core values

## Abstract

Healthcare has long been marked by the authoritative-physician–passive-patient interaction, with patients seeking help and physicians seeking to restore patients back to health. However, globalisation, social movements, and technological advancements are transforming the nature of this relationship. We aim to identify core values that influence the power dynamic between patients and healthcare professionals, and determine how to steer these interactions towards partnership, a more suitable approach to current healthcare needs. Patients with chronic diseases (10 men, 18 women) and healthcare professionals (11 men, 12 women) were interviewed, sessions transcribed, and the framework method used to thematically analyse the data. Validation was done through analyst triangulation and member check recheck. Core values identified as influencing the patient-healthcare professional power dynamic include: (A) values that empower patients (acceptance of diagnosis and autonomy); (B) values unique to healthcare professionals (HCPs) (acknowledging patients experiential knowledge and including patients in the therapeutic process); and (C) shared capitals related to their interactions (communication, information sharing and exchange, collaboration, and mutual commitment). These interdependent core values can be considered prerequisites to the implementation of the patient-as-partner approach in healthcare. Partnership would imply a paradigm shift such that stakeholders systematically examine each other’s perspective, motivations, capabilities, and goals, and then adapt their interactions in this accord, for optimal outcome.

## 1. Introduction

*“Power is not an institution, and not a structure; neither is it a certain strength we are endowed with; it is the name that one attributes to a complex strategical situation in a particular society.”*

*Michael Foucault*

A critical analysis of healthcare systems reveals that the dominance physicians have enjoyed since the inception of biomedicine has influenced the development of healthcare systems in our society. At the beginning of the twentieth century, professional organisations that governed the workings of the medical profession were developed and exclusive, high-priced licensing programs introduced. This institutionalised the existent dominance of the conventional physician, while ousting older healthcare providers such as midwives, herbalists, and emergent fields such as chiropractic, who lacked the social status, power, or money to compete [1] (pp. 286–303). The evolution of the pharmacist’s role from administrative to clinical care [2]; emergence of nurse practitioners, certified mid-wives, acupuncturists [3], radiation technologists and the development of autonomous physiotherapists independent from physicians [4], led to the recognition of other healthcare professionals (HCPs), initiating the diminishing power of physicians in healthcare. These HCPs are increasingly entrusted with responsibilities and decisions that initially were the sole responsibility of the conventional physician, for example prescribing and administering medication [5].

In the traditional physician—patient interaction, both parties view the physician as the final authority and expert in health-related matters, utilising their skills to choose the treatment most likely to restore patients to health or reduce their pain [1,6]. Their skills, technical knowledge, the trust of patients who come to them for help [7,8], and their ability to obtain available resources from the system puts the physician in a superior position [9], (p. 439). However, over the last three decades, an information revolution, social movements, and globalisation have increased patient accessibility to health information and health experts [3], empowering them through learning and exposure to different sources of information [10]. In addition, today people are increasingly aware that genetics and lifestyle (diet, exercise, sleep, and chronic stress) bear a direct impact on health, disease management, and the ability to influence one’s own health [1]. Subsequently, we see an increasing number of people with chronic diseases no longer wishing to submit to treatment without question, but preferring to manage their disease with relative autonomy. Patients are either seeking information and choosing to be actively involved [11] or they are being thrust into change by the need to manage a chronic disease [8].

These developments illustrate how healthcare systems have evolved based on conventional norms of the time—namely social behaviour and knowledge acquisition (micro level changes), which in turn have influenced changes at the macro level (structures, institutions, and systems of operation). Over time, these structures became legitimised through legislature, licensing programs, and educational requirements [1,12], reinforcing the power imbalance. This demonstrates how power, studied at both the micro (interconnected individuals) and macro (institutions and countries) levels of exchange [9] (p. 431), emanates from the bottom [12,13], but is exercised from the top, through a complex network of interactions. However, our study focuses on power shifts at the micro level within the healthcare systems and their compounding effect, which necessitates adaptive macro level changes. The increase in the number of participating actors in healthcare urges us to analyse the distribution of power within a network, as opposed to within dyadic relations (e.g., patient—physician) [14]. On one hand, power is increasingly shared among more HCPs as new positions gain autonomy, recognition, and scientific advocacy. On the other hand, increased awareness in health-related matters, coupled with choice, grants patient some of the decision making power.

Admittedly, HCPs continue to retain a predominant role. Trust in traditional relationships alone is either waning [3] or becoming non-existent as it is no longer sufficient to guarantee quality of care [6,15] in the current environment strained by limited resources [16]. Healthcare systems are responding by introducing new ways of delivering healthcare. Initially, interventions focused on the acquisition of new skills by HCPs [17]. However, HCP competences alone do not offer sustainable long-term solutions. The acquisition of skills to motivate patients to adhere to treatment would be incomplete without seeking to understand from patients why they sometimes ignore recommendations. This inclusion of the patient’s perspective is evidenced by the proliferation of micro level studies on approaches to patient care, characterised with topics such as patient engagement [18], patient-centeredness [19], patient empowerment [20,21], patient organisations, health literacy [22,23], and patient partnerships [24].

We posit that the Patient-as-Partner Approach is best suited for long-term healthcare as it challenges the traditional core values of healthcare mentioned above and guides development according to emergent norms of rebalancing knowledge, control, and power dynamics in the HCP—patient relationship. The concept proposes the recognition of patients’ experiential knowledge as complementary to HCPs scientific knowledge and proposes that the patient be considered a fully-fledged member of the healthcare team who participates in decision-making processes related to their healthcare [25,26]. Partnership therefore entails acknowledging the patient as an essential contributor to therapeutic process, and encouraging them to step into the role of a partner. Over time, patient dependency and disproportionate power, which produces inequality in relationships, will tend to gravitate toward more balanced exchanges [9] (p. 431) or partnerships. Today, the reduction of social inequalities is a priority [27], suggesting the need for new solutions and strategies in coping with changing demands. The qualitative approach, considered most suitable for initial explorative analysis was therefore used. Our study, conducted in Luxembourg, is part of a larger collaborative project (*Approche Patient-Partenaire de Soins APPS-INTERREG)* in the Greater Region (the Greater Region is the area of Lorraine (in France), Luxembourg, Saarland, Rhineland-Palatinate (in Germany), Wallonia and the rest of the French community of Belgium, and the German-speaking community of Belgium). Our aims are to identify core values of the HCP-patient interaction that influence the power dynamic, and to determine how to tip them in favour of the patient so as to steer interactions towards partnership, which would promote up-to-date and self-sustainable healthcare systems.

## 2. Materials and Methods

### 2.1. Population and Recruitment

Patients: People suffering from physical chronic diseases (cancer, diabetes, chronic kidney disease, rare diseases, cardiovascular diseases, and stroke) participated in semi-structured focus groups. Patient associations were contacted via flyers, emails, and phone calls. The committee members of these associations worked in collaboration with the research team to organise the sessions.

Healthcare Professionals: General practitioners, specialists, and nurses working with physical chronic diseases (general medicine, cardiology, nephrology, oncology, pulmonology, haematology, endocrinology, geriatric, and rheumatology) participated in individual interviews. Recruited through patient recommendations or with the help of two nurses (who presented the study to HCPs) working part time on the project in addition to their daily tasks. Both patients and HCPs were interviewed to offer a balanced point of view.

The inclusion criteria, based on a literature review conducted by a multidisciplinary scientific committee of the APPS-INTERREG project, showed that at the initial phase of disease manifestation and diagnosis, when patients experience the expression and aggravation of symptoms, their ability to act is impaired by the shock of disease onset. Gradually however, they regain their capacity for action and develop coping strategies. Patients who are members of a patient association were therefore selected to participate, as they are more likely in a stabilised phase of their healthcare journey and better able to give a more complete picture of the evolution of their interactions with HCPs and the state of partnership. Excluded were patients with mental and psychiatric chronic disorders, dementia, and HCPs who treat these chronic diseases. HCPs who treat patients with physical chronic diseases were selected. All participation was voluntary and no compensation or incentives were given.

### 2.2. Procedure and Ethical Consent

Each participating partner obtained its own ethical approval, and signed a collective scientific agreement (APPS 032-3-06-013). In Luxembourg, the principal investigator elaborated the protocol and the patient ethical consent form, to obtain ethics approval (R-AGR-3192-11), from the Institute of Research on Sociology and Economic Inequalities. The interview guide was developed and piloted in the Greater Region, to ensure face validity, check participant comprehension and test completion time. Minor adjustments were made to reflect feedback. After translation and back-translation by native experts, the interview guide was proposed in French, German, and Luxembourgish. Supplementary information (a project flyer, details of the project, funding agencies, and an official invitation letter) was given to those who wished to participate, and interview dates were set. A few days before the sessions, the interview questions, a sociodemographic form, an appointment confirmation letter, and a consent form were emailed to the participants.

### 2.3. Data Collection

Data collection and analysis took place over a 1.5-year period. Two trained interviewers conducted sessions, using a semi-structured interview guide. The principal investigator (PI) explained the goal of the study and data usage. Participants were informed that the data would be anonymised to protect their identities and that they were free to drop out at any time. They kept a signed consent form. The focus groups took place at the University of Luxembourg, in a hospital, or at a patient association centre. Each lasted 45–130 min. Individual interviews took place at the HCPs workplace and lasted 30–120 min. Sessions were audio recorded, anonymised, and transcribed. Non-French transcripts were translated and all data analysed in French. Triangulation of sources was used to ensure robust and well-developed sources. First, semi-structured research questions were developed based on the literature definitions of the patient-as-partner approach. Second, data was collected from HCPs and third party sources consisting of focus groups with people with one or more chronic diseases.

### 2.4. Data Analysis

The framework method was used to conduct content analysis, as data was homogenous. Four broad categories aimed at determining the requirements for the implementation of partnership were applied as an overarching analytic framework of analysis. These general categories, defined following an extensive literature review on patient partnership in healthcare, include: (1). Core values of patient partnership; (2). factors that facilitate partnership; (3). obstacles to patient partnership; and (4). solutions for the development and implementation of partnership. In this study, we present the results of the first of these four key dimensions—the core values of patient partnership in healthcare.

**Figure 1:**
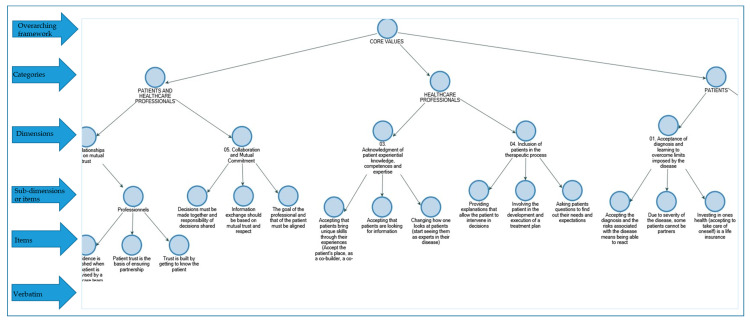
A section of the parent–child framework developed and used in the coding. Verbatim was not included as they are too detailed and voluminous to be displayed. Please see supplementary file 1 for a complete list of (74) items.

The thematic content analysis was mostly inductive, with an explorative data abstraction conducted from the specific and anecdotal to broader, more general categories. Emergent themes (categories) were all were classified under predefined dimensions (based on the literature review), therefore, the coding was also deductive in nature. This systematic coding of data into broader and specific themes and sub-themes resulted in a parent-child/tree structure as illustrated in Figure 1. The decomposition and reconstitution of the data allowed its organisation in a way that answers the research questions [28].

To ensure analytic rigour, the investigator triangulation method was used. First, the principal analyst (PA) read the interviews, thematically grouped similar verbatim together, and formulated a statement that captures the meaning of the grouped verbatim. Secondly, another analyst verified the PA coding, to minimise potential biases. As verbatim were added, categories merged or divided, and the analysts refined and reformulated the statements or list of items (see Supplementary file 1). As much as possible, the analysts used the respondents’ words to formulate the items. Differences were discussed and revised. Thirdly, an independent group of three—four research assistants went through the entire coding, and validated it, item per item. Contentious categories were discussed by the entire team and final decisions made by consensus. The interviewing, analysis, and validation (member and independent checking) processes continued iteratively throughout, until saturation. Saturation was determined after three transcripts analysed no longer yielded new ideas or themes. Our results highlight dimensions and sub-dimensions that make up core values of partnership, identified by both patients and HCPs. The narrative explanations are woven together from the resulting list of items, and the occasional example drawn from verbatim.

## 3. Results

### 3.1. Descriptive Statistics

Of 26 patient associations contacted, five (19%) participated in the study. In total 28 patients (64% women and 36% men) aged 32–79 years participated in eight focus groups. On average, they had been living with a chronic disease for 11 years, ranging from 1–36 years (Table 1). A majority of the patients (64%) had attained lower secondary as their highest level of education (Table 2).

Of 50 HCPs contacted, 23 participated in the study—12 doctors (59% men, 41% women) and 11 nurses (42% men, 58% women), with an average of 47 years old with 22 years in practice. Three HCPs had moved from full time medical practice to management (administration) and non-medical therapies such as exercise programs for patients.

### 3.2. Dimensions of Partnership

#### 3.2.1. Core Values Required of the Patient

##### Acceptance of Diagnosis and Learning to Overcome Limits Imposed by the Disease

Both patients and HCPs agreed that patients need to accept their diagnosis and the disease as a part of their lives. They reported that acknowledging and accepting the physical, cognitive, or emotional limitations of living with a chronic disease is a precondition to active involvement in one’s own care. Patients reported that taking responsibility for their health by influencing and respecting their bodies, helped them learn to overcome some limitations, make necessary changes, and make adaptations at home and at work, while learning to manage the unexpected. Views ranged from acknowledging the inability to participate in a partnership due to the severity of the condition, to eventual autonomy through acceptance.

##### Gaining (Patient) Autonomy

*Taking initiative to be active in one’s own healthcare:* HCPs noted that patients who embrace an active role are easier to involve in the therapeutic process. They identified some characteristics of patient autonomy. These include patients actively seeking HCPs with whom they get along, intervening in decision making, adapting their choices to the progression of their disease, and opting to remain independent for as long as possible. Patients stated that they considered it their responsibility to stay accountable and adhere to HCP recommendations.

*Cognitive capability, frame of mind, and level of education:* For cognitive capability, both actors stated that a patient’s ability to process information about their situation and respond was fundamental to partnership. For instance, survivors of stroke reported being cognitively impaired for several months to a few years, during which they were unable to make decisions regarding their treatment. Although HCPs agreed that with the right therapies patients often get better, they cautioned that with progressive diseases cognitive decline can only be slowed down, but not stopped.

#### 3.2.2. Core Values Required of the HCP

##### Acknowledgment of Patient Experiential Knowledge, Competences, and Expertise

Patients reported that living with a chronic disease enabled them to develop a deeper understanding of their experience of the illness and their reactions to treatment, diet, and lifestyle changes. They learnt to tell when something is wrong, developed a level of knowledge necessary to participate in decision making, and developed the confidence to ask questions. They affirmed that through experience and consultations, they developed the ability to recognise competent HCPs, and became empowered to fight to be understood and taken seriously. They suggested that HCPs could acknowledge their experiential knowledge and shift their attitude towards them by not underestimating their capacity to understand information and to make valuable contributions. Rather, they should accept that patients have access to other health experts, information about their disease, and will seek more open discussions during consultations.

Although HCPs acknowledged that patients contribute to data on the distinctive characteristics of a pathology, which offers a deeper understanding, they do not deem this as comparable to their own years of extensive professional training. They reported that this poses a challenge in seeing the patient as a partner. Nonetheless, they acknowledged that recognition of their own limits is a prerequisite to partnership, admitting to the need to continually challenge themselves. Furthermore, they expressed that the exponential progress in healthcare has and continues to change the social representation of their profession. Conversely, some concurred with patients that partnership means not underestimating the patient’s ability to understand and accepting that patient experiences with chronic diseases offer a unique perspective that merits recognition.

##### Inclusion of Patients in the Therapeutic Process

*Engaging patients in the management of their disease:* Patients communicated a need to maintain the possibility of discussing their options with competent HCPs, and of choosing their own treatment, conventional or alternative. They expressed appreciation towards HCPs who asked for their permission before starting treatment. Patients further observed that HCPs who explained and delegated responsibilities made it easier for them to participate in their own care, affirming that encouragement and involvement gradually prepared them to be more active. They acknowledged the need to monitor their own health behaviours.

*Involving patients in all stages of the therapeutic process:* Both actors declared that HCPs have the responsibility to include patients in discussions and in decision-making processes. HCPs reported that they did this by asking questions and verifying if patients had understood the information they had given them. This helped them understand patient expectations, gauge their level of engagement, and whether they were ready for a partnership. They asked questions to help them assess the impact a patient’s daily environment has on their ability to either adhere to treatment, or not. HCPs affirmed that they were able to identify resource patients willing to support other patients and tried to encourage them to motivate other patients to get involved in their own care.

#### 3.2.3. Shared Core Values

##### Giving, Sharing, and Exchanging Information

*Transmitting the right amount of pertinent information:* HCPs reported that they try to provide patients with information about their disease, treatment options, and the long-term value of therapy, which should help them in the decision-making process. They stated that usually they have to gauge how much information they share. Patients concurred, reporting that receiving too much information during a consultation can be unhelpful, as they need time to absorb it. HCPs asserted that it was important to ensure that patients had understood information correctly.

*Information sharing and explaining:* Patients expressed the importance of HCPs exchanging pertinent medical information with each other, reporting that oftentimes HCPs refused to release their records when they were seeking another opinion. Without their records, other treatments and recommendations prove complicated and expensive as they are forced to retake medical exams. Concerning treatment, patients wished that HCPs would work together, propose long-term plans, and accompany them in the therapeutic process. Patients stated that they rely on their HCPs to work collaboratively and to propose a comprehensive treatment plan as they cannot foresee potential hurdles in their treatment journey. For example, one of the patients in our study, diagnosed with breast cancer met her oncologist only once, neither knew his name, nor got the chance to discuss reconstructive surgery before her double mastectomy. Instead, she separately consulted with her family physician, the oncologist, and the plastic surgeon, and none of them presented her with a long-term comprehensive plan.

##### Communication/Relationships Based on Mutual Trust and Reciprocal Respect

*Mutual respect:* In their training, HCPs learned that respect dictated they communicate clearly and transparently with patients about their disease during consultations. For patients, respect manifests as HCPs taking them seriously from the beginning and regarding them as a whole person, and not just a disease to be treated. They wished HCPs would listen to them attentively and not make them feel small, recommending that HCPs could show empathy and by not judging or blaming them when they refuse treatment.

*Reciprocal trust:* HCPs stressed the need for trust more than patients did, emphasising that partnership anchors on patients’ trusting them, and talking with them openly. They noted that it was important for patients to be honest about their symptoms, their psychological state, adherence to recommendations, and any doubts they might have. Trust needs to be established from the beginning, and then strengthened, for instance, by getting to know the patient, and taking the time to listen to and reassure them. They noted that a patient’s confidence was boosted when cared for by a team. Patients confirmed this, reporting that they trust HCPs who consult with other experts, more than those who do not. They affirmed that reciprocal honesty and transparency builds a strong foundation for trust and gives patients the confidence to get involved.

##### Collaboration and Mutual Commitment

*Aligning HCP and patient goals:* HCPs noted that oftentimes their own and their patients’ goals were not aligned, affirming that understanding and aligning each other’s goals, and working together improved the chances of success. They highlighted that whereas they aim to treat disease, slow down its progression, and manage pain and side effects, patients are compelled by the desire to preserve their lifestyle for as long as possible, while managing their chronic disease and continuing to enjoy their social lives. HCPs reported that treatment often involves lifestyle changes, which can interfere with personal goals (maintain high energy levels, conceive, and cognitive clarity).

*Shared decision-making, responsibility, and accountability:* Both actors recognised that choosing a treatment plan was challenging and involved risks. For HCPs, it is important to present patients with options and to discuss the associated benefits and risks with them. When patients are afraid of making decisions, they usually ask a HCP to decide in their stead. However, HCPs maintained that in a partnership (characterised by trust and aligned goals), a suitable treatment plan should be mutually charted, and decisions made together. Furthermore, all parties must share responsibility, have an obligation in the follow-up, and accept accountability in whatever resulting outcome.

## 4. Discussion

**Figure 2:**
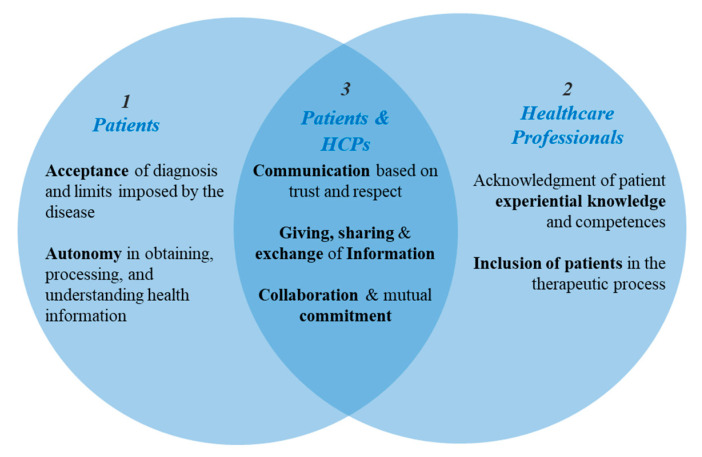
Core values that steer patients and healthcare professionals (HCP) interactions towards partnership.

Our main finding indicates that three interdependent core values fundamentally intervene on the patient-HCPs power dynamic - Values required (1) of patients, (2) of HCPs, and (3) mutual competences required of both partners (see Figure 2). These repertoire of skills, knowledge, cognitive capacities, attitudes, behaviours, and interactional styles, cultivated and applied by patients and HCPs during their interactions, can be considered prerequisites for the implementation of patient partnership in healthcare. Also known as cultural health capitals (CHC) [21,27,29,30,31], these values are leveraged to optimise the healthcare relationship. The conceptualisation of CHC relies on the Bourdieusian theory of social structure as a multidimensional field, which highlights how a purely economic approach would fail to address the fundamental hierarchical link of division between culturally dominant agents’ and the dominated ones. In practice, HCPs are still the dominant class in the healthcare field and actively participate in the early diffusion of innovations. The intermediate middle class, characterised by a ‘cultural goodwill’ *(‘bonne volonté culturelle’*), are usually ready to participate in new approaches, and therefore would be open to partnership in their healthcare. The working class and poor tend to favour traditional attitudes, balance between resistance to new tools or opportunities, and a passive attitude [27]. In this way, cultural health capital generates a hierarchical axis of domination and inequality of access between the haves and have-nots. The CHC dominant actors are able to express command in a culture of personal health and have mastered the production of legitimate understanding in the management of their health, which is valued positively. Conversely, the dominated actors generally miss potential opportunities and are forced to limit themselves to a “choice of necessity” [31] (i.e., their power is limited to the minimalist set of care assigned to them), due to a deprivation of skills and of a legitimate capacity to express their needs, feelings, and strategy in their relations with HCPs. These capitals (skills, health literacy, and legitimate expressive ability), are not only socially transmitted and differentially distributed, but are also pivotal to the ability to effective engagement and communicate during healthcare consultations [29].

We posit that to remain pertinent, core values representative of the patient—HCP interaction, must undergo an evolution from a paternalist to a partnership culture within the same healthcare system [32] (p. 16). For example, culturally-deprived patients show passive attitudes before HCPs, are more reluctant to express their choices, and are less active in the search for health strategies [30], whereas patients’ with increasing knowledge of their health condition and treatment and an ability to effectively communicate that knowledge [21] possess increased negotiation power. When HCPs demonstrate a willingness to apply this knowledge and discuss treatments options with patients, they are indicating an openness to share power with them. Our results highlight core values that need to undergo a cultural evolution in order to shift the power balance in favour of partnership.

### 4.1. Acceptance and Autonomy

In accordance with a previous study [33], our results indicate that acceptance of one’s diagnosis and autonomy are prerequisites to managing one’s disease, which is essential to becoming a partner. This extends beyond acceptance of illness, to acceptance of related pain, suffering, a life of discomfort, and limitations brought about by the disease [34]. Acceptance presents as full awareness of one’s disease and coming to terms with it [35], such that it facilitates the process of adaptation during which a patient learns to emotionally, cognitively, and physically live with the disease [36,37]. Patients who deny their diagnosis tend to surrender to the limitations imposed by the disease, fail to plan psychologically or financially [35], become more dependant, and may even become depressed [38] leading to a lower quality of life [39]. In our study, patients made needed adjustments in their personal and professional lives, demonstrating that a decline in quality of life after diagnosis can be temporary when patients take action to regain their autonomy. Indeed, adaptation is one way in which acceptance of a chronic disease manifests [37]. The attitude patients adopt towards their disease may influence their quality of life and affect their treatment outcome [34,40]. Other indicators of the ability to accept ones disease and develop autonomy in self-care include cognitive ability, level of education [8,40], and a patient’s emotional and psychological state of mind. Through education and experience, capacities can be improved and developed [41]. Indicators like level of education, as well as emotional and psychological states are not permanent, but changeable states that can be targeted to increase autonomy and therefore partnership interactions. An increase in studies on the inclusion of people with cognitive and intellectual disabilities in the healthcare process shows a trend toward supporting their self-direction as much as possible. For instance, participation in research through self-reported experience [42] and supported decision making by encouraging them to choose confidants to assist with decisions and communication [41].

The greater the acceptance, the better the adaptation and the less discomfort and depression is experienced due to the illness [36,38]. We suggest that continued adaptation leads to better disease management through experiential knowledge. For example, patients reported that with time their acquired knowledge enabled them to better understand their symptoms, and subsequently know when to take appropriate action autonomously, or when to consult an HCP. Likewise, HCPs reported that patients eventually learnt to self-administer their treatments, which allowed them the option to tailor their treatment times around their daily schedules. This self-reliance and independent decision making increases the patients negotiating capacity, contributing to the power shift from doctor and more to patient [43]. In addition to developing proactive responsibility for their health and learning to manage their disease [44], patients gain the right to choose their preferred treatment and retain control of intimate and personal decisions [43].

This has seen patients view themselves more as consumers, and the medical consultation as a transaction where they seek a better deal or shorter waiting times [3,43], which can unfortunately breed distrust. Although autonomy-inspired models, seem to protect individual rights, caution is advised as it can lead to obliviousness to the fact that a patient is still vulnerable the HCP is still dominant, and a subsequent fear of litigation operates nevertheless. Perceived distrust could therefore see HCPs withhold or restrain their inclinations to be beneficent [19,43], which would make trust a casualty of patient autonomy, thus highlighting the need for establishing new ways of building trust within the partnership.

### 4.2. Acknowledgement of Experiential Knowledge and Inclusion of Patients in the Therapeutic Process

HCPs in our study readily accepted the value of patient experimental knowledge in interventions. However, they maintained that it did not carry the same weight as their own extensive training and practice. Their values, beliefs, and behavioural norms resulting from dedicated commitment following a prolonged period of training, does by definition, make them experts in their field [3]. Knowledge and power shape social behaviours such that an extension of one means an expansion of the other [45]. Manifestly, we see HCP expertise recognised through subsequent adoption by other professionals, organisations, and policy makers. They also continue to enjoy a privileged position and status in society, and their activities are typically protected or sanctioned by the state [3]. This standing and knowledge differential makes it complicated to acknowledge and treat the patient as a partner in healthcare.

A partnership implores HCPs to challenge traditional core values that govern their interactions with patients and in some cases adapt a contrary approach. For instance, acknowledging the patient as a direct source of information by considering their experiential knowledge as complementary to their own competences [25], showing a deliberate adaptation of their attitudes, behaviours, and interaction styles in accordance with the concept of partnership. This is potentially achievable through engagement (involving patients in designing a treatment plan [19] and in decision making), compassion (aspiring to comprehend the kind of help patients need, and their understanding of the illness), and wholeness (examining the effect treatment has on patients as a whole, rather than a biological system) [6,32,46]. Moreover, involvement empowers patients to participate in healthcare delivery and governance decisions, which develops and illuminates their degree of health literacy, allowing them to manage their treatment and care [47].

The call to acknowledge gaps in their own competences and the need stay informed of new developments in their field was recognised by our HCPs respondents. Indeed, recognising one’s own shortcoming and being able to admit it is a virtue of healing. Therefore, to guarantee partnership, HCPs should know when to say “I do not know” [7], but be open to learning and walking the discovery path with the patient. The health capitals accrued by patients through experiential knowledge (lifestyle changes, self-monitoring, and imposed limitations) and intellectual knowledge (understanding reactions to medication and, learning about the disease and treatment) expand their powers [29] and reduce the knowledge differential [8]. Therefore, HCPs are not urged to change their cultural medical norms, but rather base them on tangible observable skills and behaviours that give patients a stronger voice, and give them the possibility to participate in their own healthcare. HCP compliance here symbolises an agreement to share power with the patient.

### 4.3. Communication Based on Mutual Trust and Respect

Our findings align with a previous study on patients suffering from osteoarthritis [48], which indicates that communication within a partnership is based on mutual trust and respect. HCPs emphasised the need for patients to trust them and the importance of establishing trust from the beginning, through transparency, reassurance, clarity, and getting to know their patients. Indeed, clear communication builds and strengthens trust over time [49]. Conversely, trust may be lost when patients perceive ambiguity in information given, or uncertainty about the correct course of action [50]. Traditionally, establishing trust has been the sole responsibility of the HCP, who achieves this through compassion, interest, kindness, friendliness, wit, and cheerfulness, resulting in good communication [43]. The patient simply trusts their HCP to choose the best course of treatment, to which they then adhere [6,24]. Today, the HCPs ability to establish trust can be said to be only as effective as the influence they exert on a patient’s health-related behaviours [32]. Our study showed that patients wished that HCPs would take them seriously and not regard them as a mere disease to be treated. Furthermore, they felt respected when their point of view and experience was acknowledged, which contributes to fruitful communication, patient participation [46], and builds trust.

In our study, patients reported that trust was lost when they perceived their opinions dismissed as unimportant or unscientific. Patients who desire to take care of themselves are usually interested in shared power, interdependent interactions, and a mutually fulfilling therapeutic journey, summarised as mutual participation [8]. These distinct perceptions of trust echo how different communication styles mirror different customary habits that guide our interactions [32]. To reflect partnership, core values that govern patient–HCP communication need to be redefined and to be reliant not solely on the HCP, but rather on the delicate balance between gaining and retaining mutual trust [6] and mutual respect [46], patient autonomy notwithstanding. Indeed, respect for patient autonomy is advocated to avoid communication failure and lack of trust [43]. The autonomous patient is also responsible for retaining HCP trust through honesty and transparency about their treatment, symptoms, psychological state, and any doubts, which builds trust and optimises outcome [51].

### 4.4. Information Sharing and Exchange

Although diagnostic disclosure is common in some cultures, there is no universal consensus about the quantity of information to disclose to a patient [32]. In our study, HCPs reported that they are expected to assess and adapt the amount information they give to each patient. Open questions could be used to asses and understand a patient’s situation, expectations, and perspective, and to inform HCP behaviour and attitude adaptations. HCPs could use the nature of questions or lack thereof as a sign of a patient’s capacity to understand the true nature of their situation [52] and potential patient engagement. The amount of information shared and quality of communication depends on the type of consultation, with patients reporting that diagnostic consultations are often tense, traumatic, and unforgettable. Therefore, how information is shared at this stage is critical, with poor communication lacking in sensitivity likely to lead to non-adherence and (patient) non-returns.

Today, patients expect to discuss their options during consultations [4] and for their opinions to be taken into account in the treatment plans. This attitude has ushered in the arrival of the impatient patient, one who expects increased speed in their medical care delivery, thanks to accelerated technological innovations [53]. Partnership dictates that decisions are jointly made, which presumes that both actors understand the disease and treatment. However, access to information does not automatically prepare patients for partnership. In fact, patients often find health information (oral and written medical instructions, associated treatments and health insurance forms) difficult to understand [22], which has been related to an inability to participate in decision making and to non-adherence to medical advice [23,54]. This highlights the importance the patient’s ability to not only obtain information, but also to process and understand it, highlighting intellectual capacity, as a precondition to autonomy.

Information sharing facilitates care through smooth and coordinated healthcare flow, not only between patients and HCPs, but also between different providers and healthcare settings [55]. Patients expressed frustration in the lack of medical information exchange among HCPs. Continuity of care depends on the completeness and availability of medical records [49], which helps to develop a comprehensive therapeutic plan, with short- and medium-term goals.

### 4.5. Collaboration and Mutual Commitment

Collaboration between HCPs and patients is necessary in long-term care [19,56]. Although no consensus exists on what constitutes collaboration or how to achieve it [56], our results indicate that goal disparity between HCPs and patients hinders collaboration. For instance HCPs are trained to adopt a mechanistic model of illness, rely on interventions, value emotional detachment, trust their clinical experiences [1], and relieve pain and suffering [19,43] whereas patients want to learn how to live with their disease while maintaining their job and family obligations [4]. As such, if they deem treatment as too intrusive on their ability to perform essential daily tasks, they are less likely to comply with HCP advice [54] or to disclose non-compliance to their HCPs.

Patients need to be open about personal priorities and goals that would hinder adherence to treatment. As they may not be immediately aware of these [56], they would need time to understand the complexities of treatment and make adjustments before they can evaluate the benefits and risks [36,57] and even articulate them [58]. Essential to patient participation is their ability to accurately and concisely communicate accrued knowledge [16,22]. HCPs on the other hand should favour holistic models over the mechanist approach in their interactions with patients, so as to enable some reconciliation between the core values and principles that drive each side’s actions [7] and highlight what constitutes “acceptable risk” for both parties [57]. In addition, this would facilitate continued open discussion and adjustments to the therapeutic plan, allowing for collaborative goal setting and providing shared goals to work towards [19]. When the risks and benefits are understood, and the outcome accepted by all, both parties can approach the set goals collaboratively, and with mutual commitment [8]. This has been shown to encourage patient participation and the active management of their disease [19].

## 5. Strengths and Limitations of the Study

Our sample, recruited from among patients who are members of patient associations are more stabilised in their therapeutic journey. They have more experience in participation, are likely better at managing and coping with their disease, compared to patients who are in a pre-stabilized phase in their health journey.

Sex and age have a different impact on the occurrence of chronic disease, therefore the inclusion criteria advocated for a gender balance among respondents and a heterogeneous age (from 18 years) in the composition of our sample. Our patient sample consisted of almost twice as many women as men and our youngest participant was 32 years. It is therefore possible that younger adults, in their twenties might have a different culture and exhibit different interaction patterns with their HCPs.

Since participation was on a voluntary basis, it is possible that the HCPs who chose to participate are already interested in and practising partnership, even though it is without the label. In contrast, it is not possible to know if the profile of the HCPs who declined the invitation to participate is different and if so, in what way.

The word partnership did not feature in the usual patient-HCP interactions. The term, derived from the research literature, was to an extent imposed on the participants, prompting responses associated with the terminology, potentially acting as an unintended influence on the discourse.

## 6. Conclusions 

The core values identified as essential to partnership are interdependent, influencing each other in different ways, depending on the capitals possessed by the interacting partners. For instance a patients degree of acceptance and autonomy determine their willingness and ability to adapt, collaborate, share information, and communicate honestly and effectively. On the other hand, a patient in denial will likely not make any deliberate life changes as they are “not sick”. Their communication will therefore not include an exchange, reflective of the challenges brought about by the disease. Likewise, HCPs who collaborate, share and exchange information, communicate respectfully, and build trust are acknowledging the patients experiential knowledge, inspiring their participation. However, an HCP that does not acknowledge the patient’s experiential knowledge is more likely to take a more directive as opposed to democratic interaction style. Different interactions of these core-values that can be explored, perhaps even on a continuum, where possessing few of the identified capitals places the interaction at the beginning of a potential partnership and the acquisition of more capitals from both sides, makes the interactions more equitable, and towards the desired partnership.

Partnership is not about the equal distribution of power, rather about the continuous search for balance between two types of knowledge. Experiential knowledge acquired by patients who have to learn to live with a disease for the rest of their lives and scientific knowledge, acquired through formal training, by those who seek to help. Each party needs to recognise the competencies, capabilities, and skills of the other, at different points in time. Therefore, partnership symbolises power sharing based on the patient’s acquisition of prerequisite capitals of acceptance and autonomy, and the HCPs conscious learning, unlearning, and adjustments of attitudes and skills.

The idea of partnership in healthcare implies a paradigm shift in which all partners step back from their usual traditional roles and experiences and put themselves in the other’s shoes, to try to understand and respect their perspective, motivations, and capabilities. Therefore, all actors need to make concessions to find a middle ground in which partnership can be conceived, birthed, and flourish.

## 7. Practical Implications

This study focused on the patient–HCP interaction. A next step would be to consider the structural factors in the application of our findings. For instance, the involvement of patients in HCP training has been shown to have positive effects on trainers, students training to be HCPs, and patients themselves [59]. Learning from patients leads to better understanding the patient, increases the learner’s confidence, and encourages them to talk openly with patients, even about intimate matters. HCPs in training acquire clinical examination skills taught by patients, just as well as when taught by HCP trainers [16]. HCP trainings should therefore be enriched by including patients as partners from the beginning. Patients’ experiential knowledge can therefore inform the development of better healthcare interventions [26].

Based on our results (items), we developed two partnership (Likert scale) questionnaires. One for HCPs and the other for patients. These measurement instruments will be validated and through them, we will reach more respondents, allowing us to advance and refine our findings.

The evaluation of core values status can help to assess whether actors possess the capitals necessary for partnership, as well as inform the development, training, and monitoring of these capitals in programs designed for patients, HCPs, and healthcare institutions. This would allow for the development of future healthcare systems, adapted to increasing demands while providing quality care and optimising the use of limited resources.

By interviewing patients with various chronic diseases and HCPs who manage and treat several chronic diseases, we were able to focus on convergent core values, representative of a range of diseases. Our findings are therefore transferable to multiple situations, as opposed to a single group/disease.

Including patients in the healthcare process empowers them to play an active role in their own healthcare journey and relieves pressure off healthcare systems, already burdened or likely to be in the near future.

Although not included in our selection criteria, these findings could be adapted to inform patient partnership projects among people with mental and psychiatric chronic disorders, as well as among people with cognitive or intellectual disabilities.

## Figures and Tables

**Table 1:** Descriptive statistics.

	Obs.	Mean	Std. Dev.	Min	Max	% Percent
**Patients**						
Age	28	56	11.1	32	79	
Years since diagnosis	27	11.2	9.2	1	36	
Sex	28					
Women	18					64.3
Men	10					35.7
**Healthcare Professionals (HCPs)**						
Age	23	47.4	8.1	38	62	
Years in practice	22	22.1	8	7	37	
Sex	23					
Women	12					52.2
Men	11					47.8

**Table 2:** Patients level of education.

Highest Level of Education	Freq.	% Percent	% Cum.
Doctorate or equivalent	1	3.6	3.6
Masters or equivalent	4	14.3	17.9
Bachelors or equivalent	1	3.6	21.4
Post-secondary diploma	2	7.1	28.6
Post-secondary certificate	2	7.1	35.7
Secondary school	18	64.3	100
Total	28	100	

## Supplementary Materials

The following are available online at https://www.mdpi.com/1660-4601/17/22/8458/s1, Table S1: Complete list of items generated from the qualitative data analysis.
