# Supplementary material for: Core Values that Influence the Patient—Healthcare Professional Power Dynamic: Steering Interaction towards Partnership
Source: Int J Environ Res Public Health. 2020 Nov 15;17(22):8458. doi: 10.3390/ijerph17228458 (PMC7696821; doi:10.3390/ijerph17228458)
Supplement: Supplementary file 1 [file ijerph-17-08458-s001.pdf]

Supplementary Table S1: Complete list of dimensions and items generated from the qualitative data analysis

Number of sessions = Number of times each (item) was mentioned per session (interviews & focus groups)
Number of Verbatim = Total number of times an item was mentioned (number of verbatim).
As much as possible, items were formulated based on the words used by the respondents.

PATIENT VALUES			HEALTHCARE PROFESSIONAL VALUES			MUTUAL (PATIENTS & HCPs) VALUES		
**Acceptance of Diagnosis and Learning to Overcome limits imposed by the disease**	No of sessions	No of verbatim	**Acknowledgment of patients experiential knowledge, competences and expertise**	No of sessions	No of verbatim	**Giving, sharing and exchanging information**	No of sessions	No of verbatim
Accepting ones diagnosis and illness means getting involved in the care process			***Recognizing knowledge acquired through experience with the disease and skills related the care received***			Gauging (knowing how to dose) the amount of information one gives to each patient	12	30
Acceptance of illness is a prerequisite for partnership	10	16	Accepting that patients bring unique skills through their experiences (Accepting the patient as a co-author of their therapeutic journey)	4	22	Provision of sufficiently extensive explanations to help patients intervene in decisions making	7	10
Investing in one's health (accepting to take care of thyself) is one's own life insurance	4	13	Accepting that patients will seek information elsewhere, and subsequently more open discussions	7	9	Discussing and sharing information between the healthcare team and also patients	4	15
Accepting ones diagnosis and the risks of the disease means being able to respond correctly	3	5	Acknowledging that patients provide data on the peculiarities of a pathology to offer a deeper understanding of what works and what doesn't	3	3	Discussing prospects (medium and long-term treatment plans) with patients	1	2
Learning to test ones abilities to modulate what one can do as a patient	2	5	Changing the way one views patients (starting to consider them as experts in their own right)	2	3	**Communication based on mutual trust and reciprocal respect**		
Taking charge of one's disease			Accepting that patients can recognize competent professionals through their personal experiences	2	3	***Discussions based on reciprocal trust***		
Finding information about ones illness (on the internet, and abroad)	7	13	***Recognizing the evolution of the medical culture for professionals***			Trust must be established when entering care	8	14
Becoming independent in one's healthcare is to gain some freedom	4	12	Remaining open to alternative healthcare, albeit frank in sharing ones professional opinions	5	14	Showing patients that one (HCPs) has time to listen to them, and reassuring them builds trust	7	15
Getting involved in monitoring your habits (diet, alternative methods)	3	12	Avoid positioning oneself at the top of the pyramid as the only one with pertinent knowledge	5	11	HCPs must be able to advise and to refer patients to other HCPs	3	4

Having the opportunity to choose ones treatment among available options	3	8	Acquisition of professional maturity in healthcare over time (developing ones different skill-sets)	4	5	Exchange of information should be based on mutual trust and respect	6	10
Fighting to make one's self and ones experiences heard	2	8	Offering other non-medical and non-surgical treatment options	3	13	Both actors should be honest in their interactions with each other	2	3
Being responsible for sharing experiential knowledge with HCPS, to enrich their knowledge	2	2	Acknowledging recommendations that patients bring from other HCPs / specialists	3	7	***Relationship based on mutual respect***		
Learning to deal with the unknown	2	2	**Inclusion of patients in the therapeutic process**			Taking patients seriously from the start means accepting that that they are being honest	9	21
**Gaining (patient) autonomy**			Taking time to include patients in treatment decisions	10	13	HCPs must be able to show empathy (e.g. trying to put themselves in the patients shoes)	9	16
Taking the initiative to stay active in one's healthcare			Checking if patients agree to be involved in the decision making	10	12	HCPs should make an effort to get to know the patients (background, character)	6	18
Intervene to participate actively in discussions because disease has become part of one's life	8	12	Involving the patient in the management and development of the treatment	8	16	HCPs must take the time to listen to patients	5	10
Taking responsibility manage ones disease by owning it (for as long as possible)	7	15	Integrating patients into the healthcare process, by taking their personal environment into account	7	11	HCP Communication must be transparent and clear	4	10
Getting informed and asking HCPs questions to clear doubt and gain understanding	6	10	Asking patients questions to find out about their needs and expectations	7	7	HCPs should keep emotionally distant to give unbiased judgment	2	2
Taking responsibility to adhere to HCP recommendations	5	7	Being ready to initiate partnership at any time during the therapeutic process	6	7	HCPs ought to be cautious not to guilt trip or judge patients who do not want treatment	2	2
Advancing in the management of ones pathology during the therapeutic journey (both at home and at work)	5	6	Offering patients treatment options as it is their right	5	9	**Collaboration and Mutual Commitment by both HCPs and patients**		
Finding a HCP with whom one gets along well	3	7	Identifying resource patients who could be part of the support team for other patients	4	9	Professionals must use plain language to be understood by patients	12	24
Knowing how to listen to one's body is to understand what's working and what's not working	3	6	Checking whether patients have understood the information received	3	3	Confidence is built when the patient is supervised by a healthcare team	6	11

The frame of mind, level of education and cognitive capability to gain autonomy			Leaving more initiatives to patients	3	3	Multidisciplinary healthcare teams should collaborate and work with patients to find the best solutions	5	19
Possession of the knowledge or intellectual capacity to discuss and understand ones treatments and the therapeutic process	14	18	Asking for patient's agreement / consent before starting treatment	3	3	HCPs should evaluate and adapt their language to the patient's culture, language and needs	5	10
Needed level of education or intellectual capacity to discuss and understand	6	17	***Discussing the information to be transmitted to adapt it to the skills of each patient***			Collaborate together with multidisciplinary care teams and patients to find better solutions	4	18
A sound state of mind in the patient facilitates his care	5	4	Explanations to patients must relate to treatments	13	27	**A reciprocal commitment between professionals and patients**		
Disease severity impacts the ability to partner in one's care	3	9	Checking if (health / treatment) information provided by a patient is accurate	7	9	The HCP and the patient goals must be aligned	9	14
			Showing interest to patients by adopting a partnership attitude			Decisions must be made together, and all parties held accountable	6	8
			(Know how to be a psychologist) Take time to reassure patients	4	8	Actors should consider each other equals	4	6
			Having partnership attitude gives the patient self-confidence	4	10	All actors (HCPs and patients) are responsible for patient monitoring and follow-up	4	5
			Make the effort to find out if the professionals have not yet encountered this case	2	5	The desire to interact in partnership must be accepted by all parties	4	4
